# Nuclear pore protein Nup98 is involved in replication of Rift Valley fever virus and nuclear import of virulence factor NSs

**DOI:** 10.1099/jgv.0.001347

**Published:** 2019-10-31

**Authors:** Simone Lau, Friedemann Weber

**Affiliations:** ^1^​ Institute for Virology, FB10-Veterinary Medicine, Justus-Liebig University, D-35392 Giessen, Germany; ^2^​ Institute for Virology, Philipps-University Marburg, D-35043 Marburg, Germany; ^3^​ German Center for Infection Research (DZIF), partner sites Marburg and Giessen, Germany

**Keywords:** Rift Valley fever virus, non-structural protein NSs, IFN antagonist, nuclear import, Nup98

## Abstract

The non-structural protein NSs is the main virulence factor of Rift Valley fever virus, a major zoonotic pathogen in Africa. NSs forms large aggregates in the nucleus and impairs induction of the antiviral type I IFN system by several mechanisms, including degradation of subunit p62 of the general RNA polymerase II transcription factor TFIIH. Here, we show that depletion of the nuclear pore protein Nup98 affects the nuclear import of NSs. Nonetheless, NSs was still able to degrade TFIIH-p62 under these conditions. Depletion of Nup98, however, had a negative effect on Rift Valley fever virus multiplication. Our data thus indicate that NSs utilizes Nup98 for import into the nucleus, but also plays a general role in the viral replication cycle.

Rift Valley fever virus (RVFV; genus *Phlebovirus*, family *Phenuiviridae*, order *Bunyavirales*) is a mosquito-transmitted zoonotic pathogen that is endemic in Africa. In large and devastating outbreaks it can kill thousands of farm animals and hundreds of humans [1].

The particles of RVFV are enveloped and contain three genomic RNA segments of negative or ambisense polarity, termed L, M and S, according to their relative size [2, 3]. There are four structural proteins, namely the two glycoproteins Gn and Gc that are inserted into the envelope, and the nucleoprotein N and the RNA-dependent polymerase L that encapsidate the genomic RNA segments to form the ribonucleoprotein particles (RNPs). RVFV also encodes non-structural (NS) proteins. NSm1 and NSm2 are encoded on the M segment as part of a polyprotein that also produces Gn and Gc, and NSs is encoded on the S segment by an ambisense strategy. RVFV enters the host cell via endocytosis and Gn/Gc-mediated membrane fusion. All steps of the viral replication cycle such as transcription and replication of the RNP-encapsidated genome segments as well as particle assembly and egress take place in the cytoplasm. The NSs protein, however, is present in the nucleus and forms large and dense filamentous structures [4, 5].

NSs is the major pathogenicity factor of RVFV that strongly inhibits host cell gene expression [6]. NSs achieves this by a multitude of activities such as targeting the general RNA polymerase II transcription factor TFIIH by (i) sequestering subunits p44 and XBP to prevent assembly of TFIIH, and (ii) driving proteasomal degradation of the TFIIH subunit p62 [7, 8]. Moreover, NSs recruits the RNA polymerase II suppressor SAP30 [9, 10], binds to a wide range of RNA polymerase II promoters [11] and hinders the export of host cell mRNAs to the cytoplasm by an unknown mechanism [12].

Despite the fact that most functions of NSs to counteract antiviral host responses occur in the nucleus, the mode of NSs nuclear transport has remained unclear. NSs has a relative molecular mass of about 30 kDa, a size that is well below the 40 kDa diffusion limit of nuclear pores [13]. However, NSs has a strong tendency to assemble into much larger filaments [14], and several mutant proteins were shown to have lost nuclear localization partially or entirely [4, 5, 15].

Previously, we have identified the nuclear pore protein Nup98 as the host cell interactor of RVFV NSs by affinity purification followed by MS [16]. Nup98 is a highly conserved component of the nuclear pore complex, and is exposed at the cytoplasmic as well as the nuclear periphery of the central channel [17]. Besides being part of the nuclear pore complex and participating in nuclear import, a fraction of Nup98 is intranuclear and involved in mRNA export, RNA polymerase II regulation and antiviral gene expression [17–19]. Unfortunately, despite our earlier MS results [16], we were unable to robustly demonstrate an interaction of NSs with Nup98 by co-immunoprecipitation (data not shown). Moreover, when cells were infected with a recombinant strain ZH548 bearing a C-terminally Flag-tagged NSs (rZH-CFNSs), immunofluorescence analyses did not reveal any obvious co-localization or changes in the subcellular localization of Nup98 (Fig. 1a). In addition, Nup98 levels did not change in rZH-CFNSs infection when compared to a control virus expressing a Flag-tagged fragment of MxA (rZH-FΔMx) instead of NSs, as shown by immunoblot analyses (Fig. 1b). Therefore, unlike other targets of NSs, Nup98 appears to interact with NSs only transiently and without any consequences for Nup98 localization or stability. Nonetheless, when cells were depleted of Nup98 by small interfering RNA (siRNA), the cytoplasmic fraction of NSs increased [Fig. 2a (upper and middle panel) and Fig. 2b]. However, the nuclear target of NSs, TFIIH-p62, was still degraded, probably by the residual NSs in the nucleus. Importantly, when the nuclear pore protein Nup62 was removed by siRNA as a control, NSs located mostly to the nucleus, although a certain increase of the cytoplasmic signal was also observed [Fig. 2a (lower panel) and Fig. 2b]. Nup62 is located in the central channel of the nuclear pore [20]. Like the more peripheral Nup98, it associates with the importin alpha/beta complex and mediates import of proteins [21]. Nonetheless, NSs nuclear import appears more dependent on Nup98 than on Nup62. Moreover, the slight impairment of NSs import could possibly also be explained by the observation that Nup98 levels can be affected by siRNA depletion of Nup62 (Fig. 2c).

**Fig. 1.**
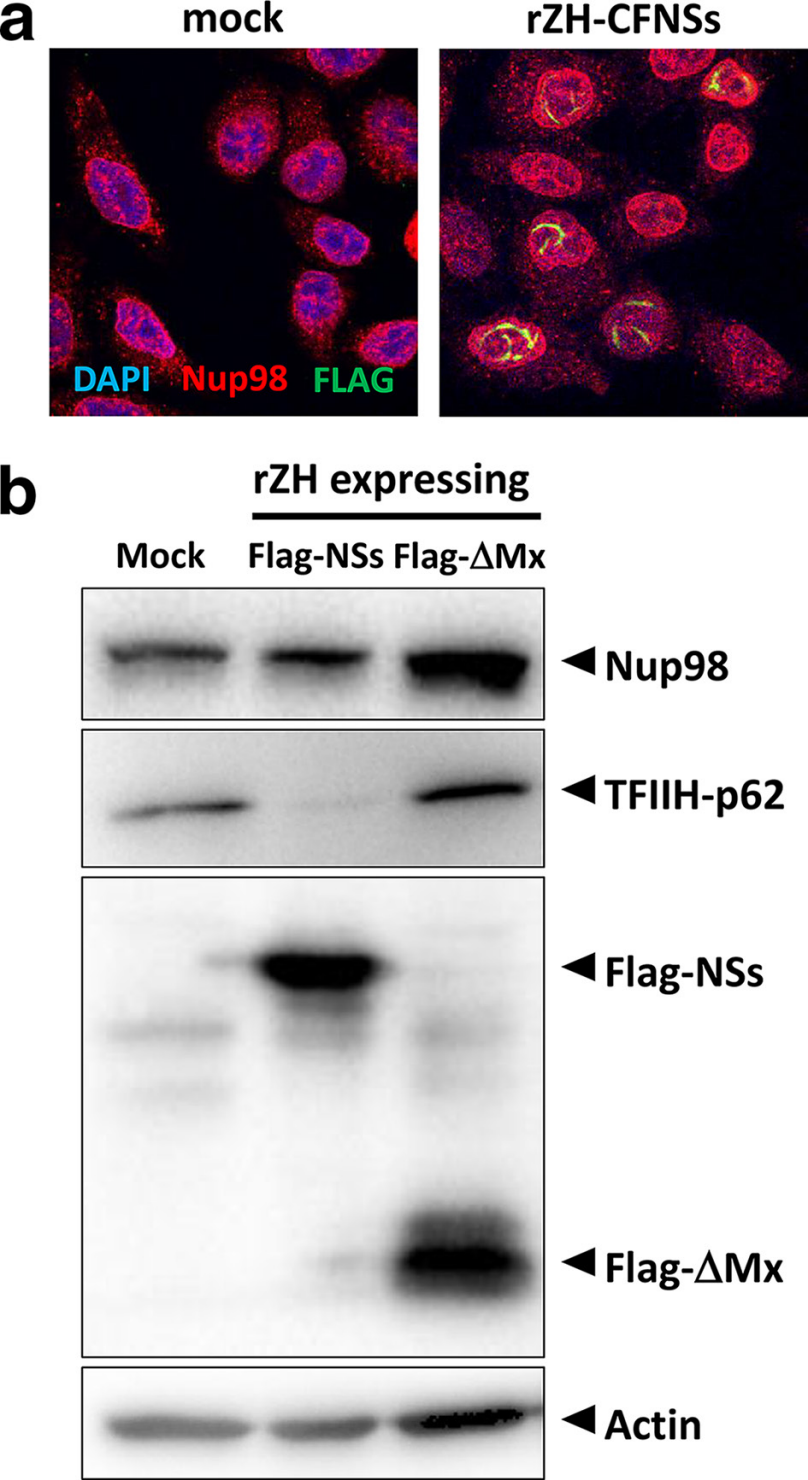
Nucleoporin Nup98 in the presence of RVFV NSs. HeLa cells were infected with recombinant RVFV expressing a C-terminal Flag tag (rZH-CFNSs [7]) at an m.o.i. of 3. After an incubation period of 6 h, cells were fixed with 4 % paraformaldehyde in PBS and permeabilized with 0.5 % Triton X-100 in PBS, and immunostained for Flag-tagged NSs (Anti-Flag M2, 1 : 1000, green channel) and Nup98 (Nup98 C39A3, 1 : 500, red channel) by confocal microscopy. (b) Immunoblot analysis. HeLa cells were left uninfected (mock) or infected with rZH-CFNSs or a control virus expressing a Flag-tagged N-terminal fragment of MxA (rZH-FΔMx [7]) at an m.o.i. of 5, and 16 h later were subjected to immunoblot analysis using antibodies against Nup98 (Nup98 C39A3, 1 : 500), TFIIH-p62 [GTF2H1 (ab55199), 1 : 1000], Flag tag [Anti-Flag (F7425), 1 : 2000] and cellular actin as a loading control (β-Actin 8H10D10, 1 : 1000).

**Fig. 2.**
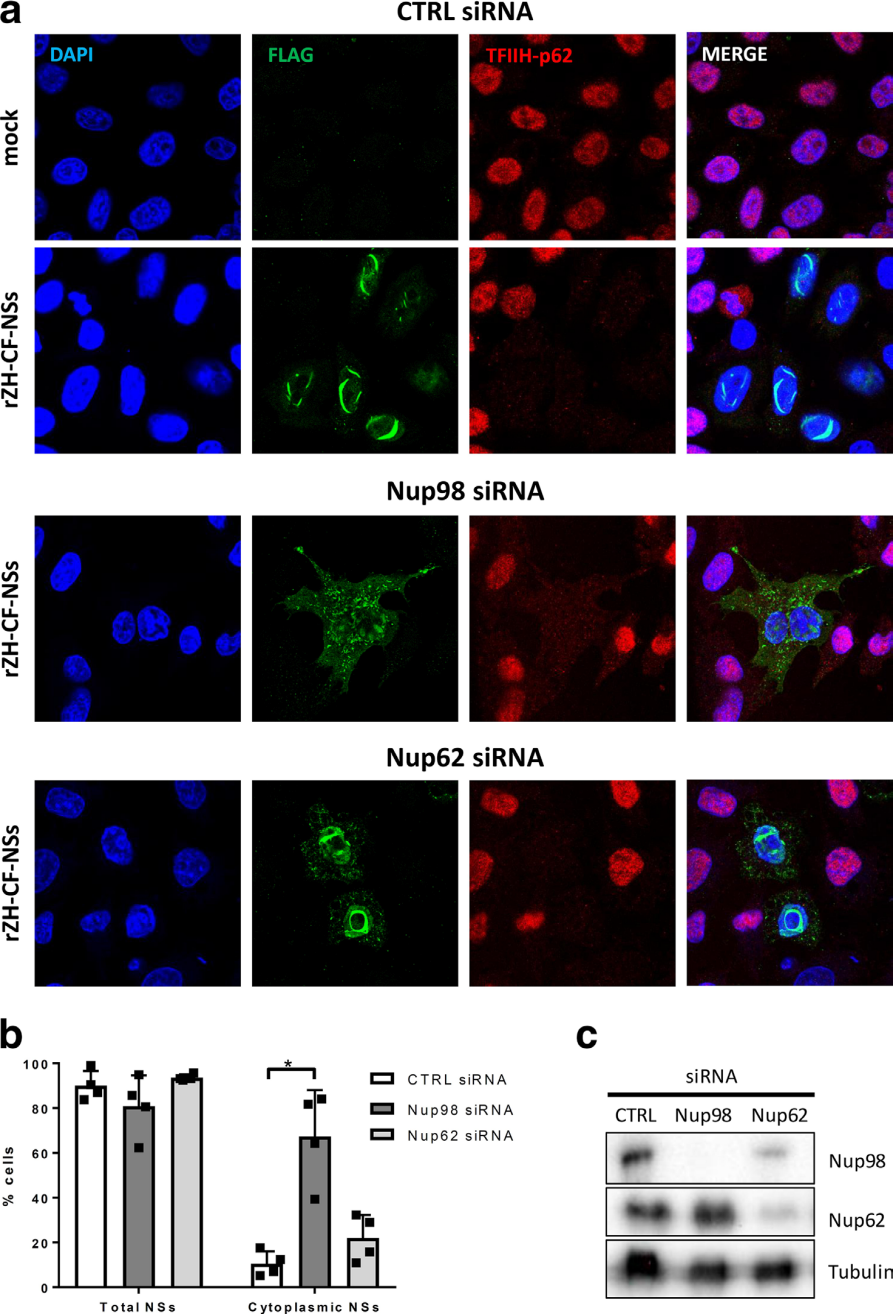
Effect of Nup98 depletion on nuclear import of NSs. HeLa cells with specific siRNA knockdown of Nup98 or Nup62 were infected with recombinant RVFV expressing Flag-tagged NSs (rZH-CFNSs) or the ΔMx control (rZH-FΔMx) at an m.o.i. of 3. After an incubation period of 24 h, cells were fixed, permeabilized and analysed for the presence of Flag-tagged NSs [Anti-Flag (F7425), 1 : 1000, green channel] and TFIIH-p62 [GTF2H1 (ab55199), 1 : 500, red channel] by confocal microscopy (a). (b) Quantification of microscopy images. In four biological replicates, around 100 infected cells per replicate were monitored for the presence of NSs and the cytoplasmic localization of NSs. *P<0.05 (Student’s paired *t*-test). (c) Knockdowns were verified by immunoblot analysis. The knockdown was achieved by two-fold reverse transfection with control siRNA (AllStar Negative Control; Qiagen) as well as validated pools of four siRNAs (Qiagen) against mRNAs for Nup98 (GeneSolution GS4928) and Nup62 (GeneSolution GS23636) as previously described [7]. Quantitative reverse-transcriptase PCR showed mRNA reduction to be at least 90 % (data not shown). One day after the second transfection cells were infected as described above. Antibodies for immunoblot were anti-Nup98 C39A3 (Cell Signaling, diluted 1 : 1000), anti-Nucleoporin p62 (BD Transduction Laboratories, diluted 1 : 4000) and anti-beta tubulin (Abcam, diluted 1 : 1000).

To investigate the importance of Nup98 for RVFV infection, we measured virus yields under conditions of Nup98 depletion. As shown in Fig. 3 (a), titres of the wt strain rZH548 dropped by almost 1 log_10_ step in the absence of Nup98, but not when Nup62 was depleted. Surprisingly, the naturally NSs-deleted strain Clone 13 was also reduced upon Nup98 depletion, although values did not reach significance levels. Thus, RVFV may require Nup98 for a general function in virus replication. Moreover, Nup98 depletion did not restore IFN induction by wt RVFV (Fig. 3b), in line with the still intact TFIIH-p62 destruction by NSs under these conditions (see Fig. 2c). However, a possible rescue of IFN induction imposed by Nup98 depletion might be hidden due to a general defect in nuclear import of transcription factors for IFN-β promoter activation. Indeed, the strong IFN induction by the NSs-deleted strain Clone 13 was reduced when Nup98 was absent, although statistical significance was not reached (see Fig. 3b). Reduced IFN induction could be due to the mentioned transport defect by Nup98 depletion, but is also expected based on the role of Nup98 in antiviral gene expression [19].

**Fig. 3.**
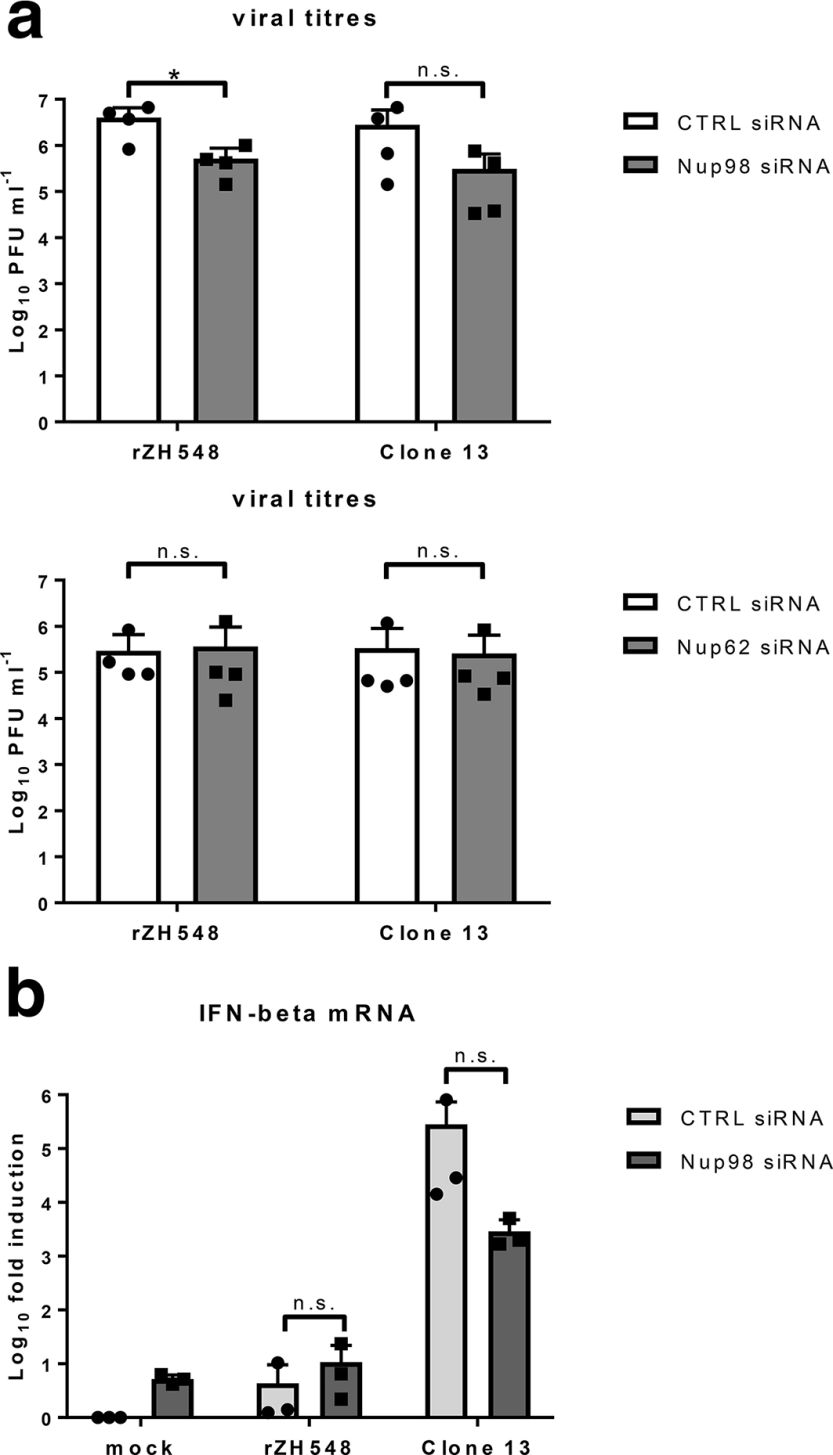
Influence of Nup98 on RVFV infection and IFN induction. HeLa cells were infected for 24 h with recombinant ZH548 (rZH548) or the naturally NSs-deleted strain Clone 13 at an m.o.i. of 10, and analysed for (a) virus yields by plaque assay or for (b) IFN induction by RT-qPCR, as described previously [7]. Results from four (a) and three (b) independent experiments are shown. *P<0.05; n.s., not significant (Student’s paired *t*-test).

Our findings indicate that Nup98, one of the most conserved nucleoporins and an important player in cytoplasmic–nuclear transport [17, 22], is required by RVFV for two independent functions. First, Nup98 is involved in the nuclear import of NSs. Nup98 is known to act as a cytoplasmic docking site for nuclear transport substrates via its unique GLFG repeat domain, as opposed to the FG repeats of the other nucleoporins [17, 23]. The GLFG repeat region is known to bind to import cargo [17]. Interestingly, both NSs and nucleoporins contain intrinsically disordered regions that have the tendency of multivalent, low-affinity interactions [14, 22], perhaps explaining the role of Nup98 in NSs import on the one hand and the lack of a robustly demonstratable interaction on the other. Unlike many other NSs interactors [7, 9, 10], Nup98 is not consumed or dislocated over the course of infection. Second, the multifunctional Nup98 may execute an NSs-independent role in the viral replication cycle, perhaps the nuclear export of co-factors required for replication in the cytoplasm, or the expression of host genes required for virus replication. Nup98 is an IFN-stimulated gene and confers antiviral activity [19, 24]. It is therefore targeted by several viruses, either by interaction (vesicular stomatitis virus [24, 25]), phosphorylation (cardiovirus [26]), degradation (influenza A virus [27]) or specific cleavage (poliovirus [28]). Our findings that RVFV does not target or modify Nup98 in a comparable, direct manner might be explained by the fact that it is required for NSs import and viral replication.
